# Burden and characteristics of Respiratory Syncytial Virus-associated respiratory tract infections in adult patients in the winter season 2023/2024 at the conservative emergency department of the university hospital in Dresden

**DOI:** 10.1186/s12985-025-02692-z

**Published:** 2025-03-17

**Authors:** J Ronczka, S von Bonin, A Laubner, K Hochauf-Stange, M Rank, M Kolditz

**Affiliations:** 1 Medical Department I, Division of Pneumology, University Hospital Carl Gustav Carus, TU Dresden, Dresden, Germany; 2 East German Lung Center, Dresden-Coswig, Germany; 3 Medical Department I, Division of Emergency Medicine and Intensive Care, University Hospital Carl Gustav Carus, TU Dresden, Dresden, Germany; 4 Institute of Medical Microbiology and Virology, University Hospital Carl Gustav Carus, Medical Faculty, Technische Universität Dresden, Dresden, Germany; 5 Institute for medical informatics and biometry, Technische Universität Dresden, Dresden, Germany

**Keywords:** RSV, Respiratory tract infections, Adults, Risk, Comorbidities

## Abstract

**Introduction:**

The burden of Respiratory Syncytial Virus (RSV) associated adult emergency department visits in comparison to other respiratory viruses like Influenza and SARS-CoV-2 remains less studied.

**Methods:**

We performed a prospective observational study to describe prevalence, severity and risk factors of RSV infection, proven by polymerase chain reaction from nasopharyngeal or pharyngeal swabs, in consecutive adult patients presenting to the emergency ward of the University Hospital Dresden with a working diagnosis of acute respiratory tract infection during the winter season between October 1st 2023 and April 15th 2024.

**Results:**

1764 adults (56.3% male) between 18 and 101 years old (median age 69 years) were included in the analysis. 477 patients (27.1%) tested positive for viral infection; 284 (16.2%) with SARS-CoV-2 (median age 79 years), 147 (8.4%) with Influenza A or B (median age 56 years) and 38 (2.2%) with RSV A or B (median age 79 years). In 8 patients (0.5%) a co-infection with two viruses was detected. In the RSV cohort any oxygen support was significantly higher (63.2%) compared to the Influenza (34.0%, *p* < 0.001) and SARS-CoV-2 (41.5%, *p* = 0.012) cohorts. In-hospital mortality was considerable especially for RSV with 7.9% compared to Influenza (2.7%, *p* = 0.138) and SARS-CoV-2 (5.6%, *p* = 0.580).

**Conclusion:**

RSV was less frequent in adults presenting to the emergency department during the 2023/24 season compared to SARS-CoV-2 and Influenza, but patients needed a higher level of respiratory support. Also, in-hospital mortality was considerable, making RSV-infections a relevant pathogen in adult patients presenting with respiratory tract infection to an emergency department.

**Supplementary Information:**

The online version contains supplementary material available at 10.1186/s12985-025-02692-z.

## Introduction

Growing evidence demonstrates a high burden of acute respiratory tract infections caused by respiratory syncytial virus (RSV) not only in children, but also in older adults with chronic pulmonary or cardiovascular comorbidities. A recently published review indicated an incidence of symptomatic RSV infection between 3 and 7% in older adults each year, which lead to hospital admission in 12% and represents 2.6–6.7% of all respiratory tract infections (RTI) related hospital admissions among older adults [1]. Based on a 2019 global systemic review and meta-analysis, there are estimated 3 million annual RSV cases, 270.000 RSV-associated hospitalizations and 20.000 RSV-related in-hospital deaths in adults aged > 60 years in Europe [2].

Recent German data confirmed a high incidence of RSV hospitalizations especially in the elderly and demonstrated high rates of oxygen need, intensive care unit (ICU) admission and mortality compared to SARS-CoV-2 or Influenza [3, 4]. Furthermore, a cross-sectional study over 5 RSV seasons estimated a prevalence of acute cardiac events of 22.4% in patients older than 50 years and hospitalized for a RSV-infection [5].

Due to the lack of therapeutic options for RSV infection to date, the focus is currently on prevention with recent vaccine developments showing over 80% efficacy in preventing RSV infections in the elderly [1]. To inform vaccine recommendations, ongoing RSV surveillance in adults at risk is necessary. As there is considerable seasonal variation of RSV incidences with data showing particularly high rates during the 2022/23 winter season and still little data on the incidence of acute lower respiratory tract infections caused by RSV in adults presenting to hospital emergency departments in Germany, ongoing data on following seasons remain crucial [3, 4].

We hereby present the results of a prospective study aiming to identify RSV incidence in comparison to SARS-CoV-2 and Influenza in consecutive adults aged 18 years or older and presenting to the conservative emergency department of our university hospital due to symptoms of an acute respiratory tract infection in the winter season 2023/2024.

## Methods

### Study design and study population

We performed an investigator initiated, monocentric, prospective observational study in consecutive adult patients presenting to the conservative emergency ward of the University Hospital Dresden with a working diagnosis of acute respiratory tract infection during the post-SARS-CoV-2 pandemic season between 1st October 2023 and 15th April 2024. Study inclusion was triggered automatically by the decision to order SARS-CoV-2 or triple PCR- test on SARS-CoV-2, Influenza and RSV from nasopharyngeal or pharyngeal swabs by the attending emergency department physician due to patient symptoms indicating any differential diagnosis of acute respiratory tract infection without definition of specific symptoms. In the case of multiple presentations during the study period, patients with positive PCR-testing for the same viral infection were included a second time only after 4 weeks. In patients presenting a second time within four weeks only the first admission was counted.

**Fig. 1:**
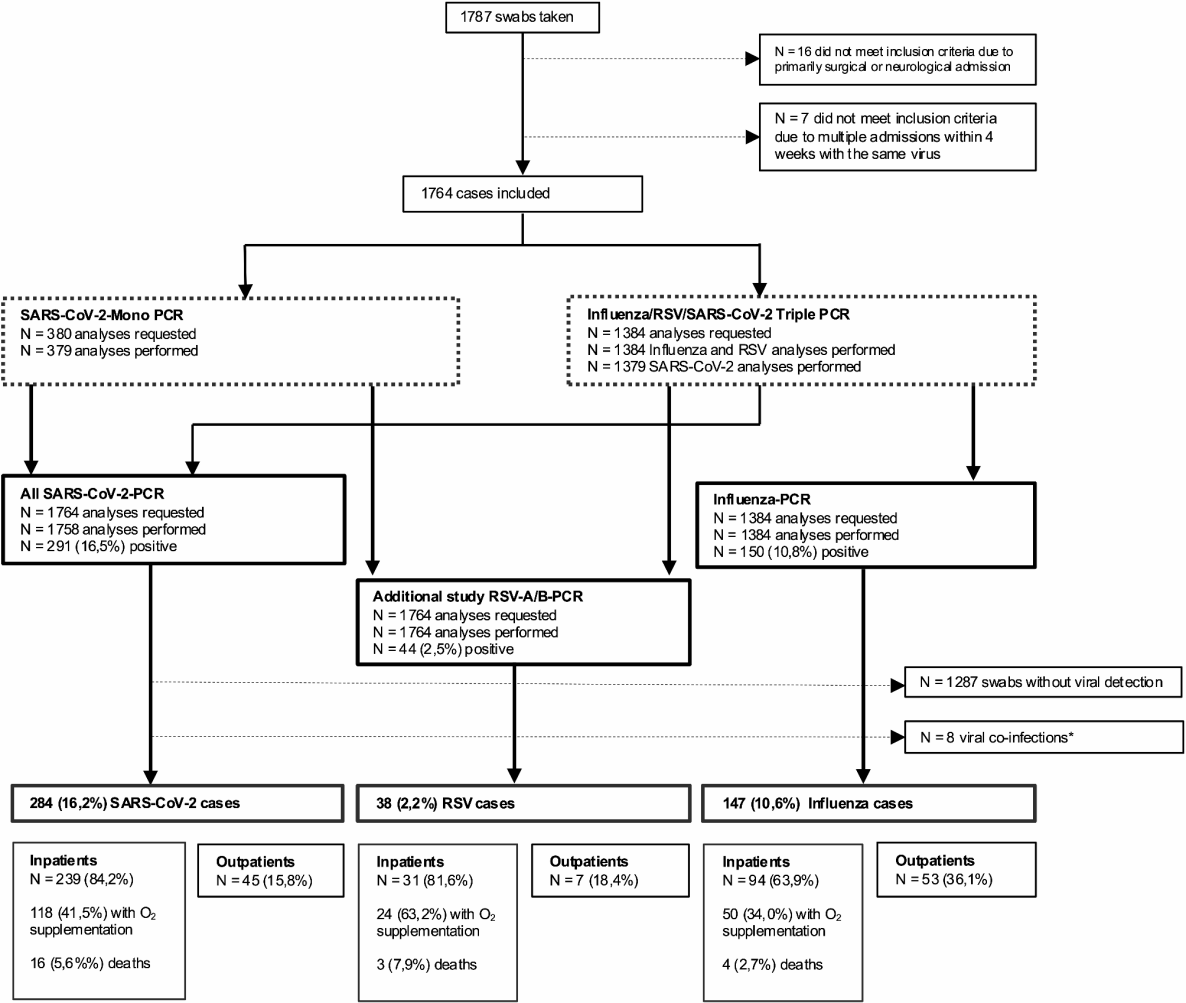
Study flow chart. * Co-infections: 5x RSV + SARS-CoV-2; 1x RSV + Influenza; 2x SARS-CoV-2 + Influenza

As shown in the study flow chart, Fig. 1, all included patients were then additionally tested for RSV by PCR (RealStar^®^ RSV RT-PCR Kit 3.0 Fa. Altona Diagnostics) from the same nasopharyngeal or pharyngeal swabs. Test results that showed an inhibition due to inhibitors in the reagent could be clarified by the initial triple PCR-testing. All PCR results were technically and medically validated by the institute of Medical Microbiology and Virology.

Demographic and clinical data such as age, gender, comorbidities, respiratory support, length of stay and outcome were retrieved from the patient’s electronic file. The Charlson Comorbidity Index (CCI) was calculated on the basis of ICD-10 coded discharge diagnoses as recently published [6]. Acute cardiac events were defined as having at least one ICD-10-Code at discharge, indicating an acute cardiac event such as unstable angina pectoris, acute myocardial infarction, acute heart failure, myocarditis or similar diagnosis. ICD-10 codes were adapted from previous studies and modified according to German ICD-10 [see Table 1, supplementary material] [5, 7].

As primary outcome we defined the incidence of RSV infections in the included study patients.

Secondary outcomes were incidence of SARS-CoV-2- and Influenza infection and parameters of disease severity including levels of respiratory support, length of hospital stay, in hospital mortality, and risk of cardiac events depending on gender, age and comorbidities.

At the time the study was conducted, RSV vaccines for adults were approved by the European Medicines Agency but not yet recommended by the Standing Committee on Vaccination (STIKO) of Germany and not reimbursed, so that no relevant immunization rate against RSV in adults must be assumed.

### Statistics

To describe the dataset, mean values, standard deviation, medians and quartiles were computed by using Software IBM SPSS Statistics, Version 29.0.0.0. The group comparisons in the form of t-tests were carried out using the same software. We employed parametric tests, although normality assumptions were not always met entirely, because sample sizes were above 30.

In order to shed more light on the differences between the three main infection groups with respect to the clinical outcomes, we applied multiple logistic regression models, including the following independent variables: type of infection, Charlson Comorbidity Index, gender and age. The binary outcome variables were respiratory support, in-hospital mortality and the occurrence of acute cardiac events respectively. Note that for each target variable a separate model was fit that was taking into account only patients that tested positive on one of the viruses of interest. From the model results, we computed the odds ratio (OR), 95% CIs, and P values using a type I error rate of 5% for all analyses. Graphics were created by using RStudio (v2023.09.1 + 494).

## Results

### Population characteristics, clinical presentation and outcomes

Out of all patients (*n* = 1787) presenting to the emergency department in the defined study period and selected for PCR-testing, 1764 (56.3% male) patients between 18 and 101 years old (median age 69 years), remained eligible for the analysis (Fig. 1).

From 477 patients (27.1%) testing positive for any viral infection, in 8 patients (0.5%) a co-infection with two viruses was detected (median age 66 years, 7 inpatients; no ICU admission or mortality). They were excluded from the analyses. From the remaining cases with one virus detected, 284 (16.2%) had a SARS-CoV-2-infection, 147 (8.4%) had an infection with Influenza A or B and 38 (2.2%) had an RSV A or B- infection (Fig. 1).

Clinical and demographic characteristics and outcomes of the patients according to different infection status are shown in Table 1.

**Table 1:** Patient characteristics according to viral infection status

Characteristics	All swabs, *n* = 1756, excluding *n* = 8 viral co-infections, *p* < 0.05 = bold						
	PCR-negative swabs	RSV A and B	Influenza A and B	*p-value* *(RSV vs.* *Influenza)*		SARS- CoV-2	*p-value* *(RSV vs. SARS-CoV-2)*
	*n* = 1287	*n* = 38	*n* = 147			*n* = 284	
Males, n (%)	716 (55.6)	22 (57.9)	78 (53.1)		*0.596*	171 (60.2)	*0.785*
Age, *years* *(median; quartils)*	68 (49;81)	79 (69;86)	56 (38;77)		***< 0.001***	79 (63;85)	*0.467*
CCI, *score* *(median; quartils)*	1 (0;3)	2 (1;3)	1 (0;2)		***0.003***	1 (0;3)	***0.023***
**Respiratory Support**							
Oxygen only, n (%)	476 (37.0)	24 (63.2)	50 (34.0)		***< 0.001***	118 (41.5)	***0.012***
nHFOT, n (%)	32 (2.5)	2 (5.3)	2 (1.4)		*0.142*	11 (3.9)	*0.684*
NIV, n (%)	63 (4.9)	1 (2.6)	4 (2.7)		*0.976*	12 (4.2)	*0.640*
Invasive ventilation, n (%)	39 (3.0)	0	4 (2.7)		*0.307*	3 (1.1)	*0.526*
**Admission**							
Outpatient, n (%)	345 (26.8)	7 (18.4)	53 (36.1)		***0.039***	45 (15.8)	*0.686*
Inpatient, n (%)	942 (73.2)	31 (81.6)	94 (63.9)		***0.039***	239 (84.2)	*0.686*
ICU-admission, n (%)	106 (8.2)	0	13 (8.8)		***0.058***	12 (4.2)	*0.198*
Length of stay, only inpatients, *days (median; quartils)*	9 (4;16)	8 (6.5;13)	8 (4;12)		*0.164*	7 (4;12)	*0.544*
**Outcome**							
Death, n (%)	88 (6.8)	3 (7.9)	4 (2.7)		*0.138*	16 (5.6)	*0.580*
Acute cardiac event, n (%)	253 (19,7)	12 (31.6)	13 (8.8)		***< 0.001***	47 (16.5)	***0.024***

P-values were determined with t-test. CCI = Charlson Comorbidity Index; nHFOT = nasal highflow therapy, NIV = non-invasive ventilation

Age differed significantly in patients with RSV in comparison to patients with Influenza, but there was no significant difference to patients with SARS-CoV-2.

**Fig. 2:**
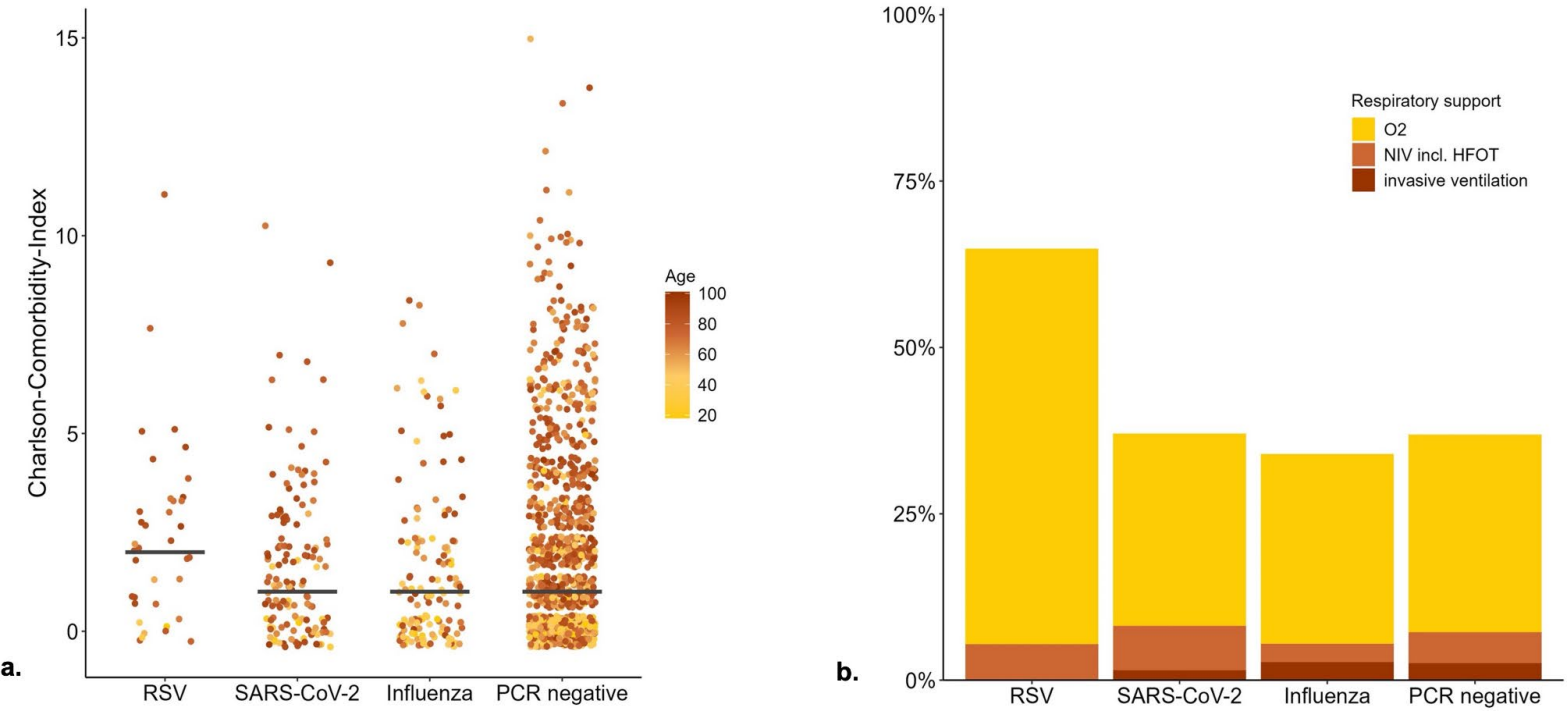
**a.** Distribution of age and comorbidity according to viral infection. **b.** Respiratory support according to viral infection

Also, Charlson Comorbidity Index was significantly higher in the RSV group than in both other groups. Both characteristics combined are shown in Fig. 2a.

In the RSV cohort oxygen administration, as shown in Fig. 2b, as well as the occurrence of acute cardiac events was significantly higher compared to the Influenza and SARS-CoV-2 cohorts.

In terms of outpatient and inpatient management, patients with RSV and SARS-CoV-2, were hospitalized more frequently than patients with Influenza, while there were no significant differences in length of stay and in-hospital mortality between all three groups.

As there were significant differences in age and comorbidities between the viral groups, we added analyses stratified for age and comorbidities. In patients ≥ 60 years of age we still found a significantly higher oxygen need in the RSV group compared to the SARS-CoV-2 (*p* = 0.004) or influenza group (*p* = 0.040). Regarding acute cardiac events, a significant difference between RSV and influenza-infected patients aged 60 years or older (*p* = 0.037) remained, while there was no significant difference between RSV and SARS-CoV-2 in this age group (*p* = 0.056). The results are shown in Table 2a in the supplemental material.

If all 3 infection groups were compared on the basis of existing comorbidities (CCI $\:\ge\:$ 1), the RSV cohort continued to show significantly higher oxygen requirement and rate of acute cardiac events compared to patients with SARS-CoV-2 (*p*= 0.015 and *p* = 0.049) or influenza infection (*p* = 0.036 and *p* = 0.030) (Table 2b, supplemental appendix).

After multivariable analysis of all PCR positive cases including type of virus, age, gender and CCI, RSV-infection remained significantly associated with need of oxygen supplementation compared to SARS-CoV-2 infection [OR (95%CI): 2.27 (1.09–5.0), *p* = 0.032] or Influenza infection [OR (95%CI): 1.67 (0.74, 3.85), *p* = 0.200].

The risk of a cardiovascular event was approximately twice as high with an RSV infection as with an influenza infection [OR (95%CI): 2.08 (0.76, 5.56), *p* = 0.150]. Even though this effect was not significant, a trend can be recognized that could be confirmed by a bigger study cohort. In addition, it was again evident that the risk of cardiovascular events also increased significantly with an increase in the Charlson Comorbidity Index [OR (95%CI): 1.35 (1.20–1.53), *p* < 0.001] and age [OR (95%CI): 1.05 (1.02–1.07), *p* < 0.001 ]. Results are shown in Table 2.

**Table 2:** Logistic regression results: factors influencing disease severity

	Respiratory support			Acute cardiac event			Death		
**Characteristic**	**OR** ^*^	**95% CI** ^*^	**p-value**	**OR** ^*^	**95% CI** ^*^	**p-value**	**OR** ^*^	**95% CI** ^*^	**p-value**
***Infection***									
RSV vs. SARS-CoV-2	2.27	(1.09–5.0)	***0.032***	1.72	(0.72–3.85)	*0.200*	0.12	(-2.7–3.0)	*> 0.900*
RSV vs. Influenza	1.67	(0.74–3.85)	*0.200*	2.08	(0.76–5.56)	*0.150*	0.49	(-2.7–3.6)	*0.800*
**Gender**									
Male vs. female	1.25	(0.83–1.88)	*0.300*	0.92	(0.53–1.60)	*0.800*	0.17	(-1.4–1.7)	*0.800*
**Age**	1.04	(1.03–1.06)	***< 0.001***	1.05	(1.02–1.07)	***< 0.001***	0.05	(0.0–0.10)	*0.053*
**Charlson Comorbidity Index**	1.20	(1.08–1.34)	***< 0.001***	1.35	(1.20–1.53)	***< 0.001***	1.10	(0.67–1.4)	***< 0.001***

^*^CI = Confidence Interval, OR = Odds Ratio

## Discussion

The main findings of our prospective observational study can be summarized as follows: [1] PCR-detected RSV-infection was present in 2.2% of adult patients presenting to our conservative emergency department with symptoms of a respiratory tract infection and with an indication for viral testing as per the treating physician in the winter season 2023-24, [2]adults with RSV had more comorbidities than those with Influenza or SARS-CoV-2 and were older than patients with Influenza, and [3] RSV-infection was independently associated with oxygen need and related to a high hospital admission rate of 81%, occurrence of acute cardiac events in 32% and hospital mortality of 8%.

There were significant differences between patients with RSV infection and those infected with SARS-CoV-2 or Influenza in terms of age, comorbidities, as well as oxygen requirements, hospital admission and acute cardiovascular events during hospitalization in our study. The association of RSV with higher need of oxygen requirement compared to Influenza even remained significant after inclusion of age and comorbidities in the model.

A retrospective observational study from Italy from the winter season 2022/2023 also showed a higher age, higher Charlson comorbidity Index and higher rate of heart failure in patients with RSV compared to Influenza [8].

The association with a higher oxygen need found by our data was also shown by a retrospective study from Rosenheim, Germany, where low flow oxygen therapy was indicated in 82.8% of patients suffering from an RSV infection in the winter season 2022/2023 [3].

Of note, acute cardiovascular events occurred in one third of all RSV infected patients. The association of acute viral RTI with cardiovascular complications is well established and contributes to the vaccine-preventable disease burden especially in Influenza [9]. Therefore, it seems remarkable that in our cohort acute cardiac complications occurred even more frequently in RSV compared to Influenza and SARS-CoV-2. Similar data were provided by a large systematic literature review published in 2024, which evaluated studies with RSV infections compared to RSV-negative flu-like illness, influenza and parainfluenza infections between 1990 and 2019. The risk of cardiovascular events in the RSV group was reported to be 1.4 times higher [10].

Additionally, in an analysis of U.S. health insurance data, collected over a 12-year period including more than 175,000 cases, the rate of acute heart failure in RSV infections was also reported to be 18% in adults aged 60 years and older [1].

Altogether these results confirm the relevant disease burden of RSV infections especially among older and comorbid adults with considerable associated morbidity and mortality, confirming the need of effective prevention strategies by vaccination in this vulnerable patient group.

The strength of our prospective study was the inclusion of all consecutive patients with physician deemed indication for respiratory viral testing, thereby avoiding any selection bias. Limitations include the monocentric study setting and an overall lower number of cases of RSV infections in the evaluated season. Viral testing was performed at the discretion of the emergency physician and not based on a prespecified protocol, which might bias the findings. Additionally, our results rely on PCR testing of nasopharyngeal or pharyngeal swabs only and therefore might underestimate the RSV burden, as it has been shown that collecting not only nasopharyngeal swabs but also sputum and saliva would increase the accuracy of testing up to 2.6 fold [1]. Additionally, studies showed a possible underestimation of virus detection by PCR testing in patients presenting to the emergency department with a relevant delay after symptom onset, which could lead to an underestimation of the viral burden [11].

We cannot rule out that viral testing was performed more often in seriously ill patients which could bias the rate of hospitalization and oxygen need. However, our relatively high admission rates especially in RSV and SARS-CoV-2 infected patients probably are associated with the high age and comorbidity burden as well as high rates of oxygen dependency of these patient groups, but still are comparable to published data from Tennessee in 2014 that showed a hospitalization rate of 75% (24 out of 32 patients) in adult patients with an RSV infection [12].

We did not include data on imaging, additional microbiology results or agreed treatment limitations in our analysis which might bias outcome results.

In addition, the immunization status of the included patients was unknown which could lead to a lower burden of influenza and SARS-CoV-2 in relation to RSV cases because the study was conducted prior to the possibility of widespread use of RSV vaccines in Germany.

Due to our study design, in which any PCR testing for SARS-CoV-2 or Influenza triggered study inclusion, only 1384 out of 1764 patients were tested for Influenza, which might underestimate the rate of Influenza; however, additional Influenza testing was triggered by German epidemiology data and all included patients were tested for SARS-CoV-2 and RSV.

## Conclusion

Our study data revealed a relatively lower rate of RSV infections during the 2023-24 winter season among consecutive adults presenting to a conservative emergency department compared to SARS-CoV-2 and Influenza. However, it highlights the relevant morbidity and mortality associated with RSV, particularly in older patients and those with comorbidities, who demonstrated a high risk of oxygen requirements, acute cardiovascular events and a substantial in-hospital mortality. These results support the need of viral testing among adults with a differential diagnosis of a respiratory tract infection in emergency departments, a need for further research into treatment options, and the effective implementation of prevention by vaccinating patients at risk.

## Electronic supplementary material

Below is the link to the electronic supplementary material.

Supplementary Material 1

## Data Availability

The data that support the findings of this study are available from the corresponding author upon reasonable request.

## Abbreviations

RSV: Respiratory Syncitial Virus
PCR: Polymerase chain reaction
RTI: Respiratory tract infection
ICU: Intermediate care unit
CCI: Charlson Comorbidity Index
OR: Odds ratio
CI: Confidence interval
U.S.: United States (of America)
