# Supplementary material for: Burden and characteristics of Respiratory Syncytial Virus-associated respiratory tract infections in adult patients in the winter season 2023/2024 at the conservative emergency department of the university hospital in Dresden
Source: Virol J. 2025 Mar 17;22:76. doi: 10.1186/s12985-025-02692-z (PMC11912720; doi:10.1186/s12985-025-02692-z)
Supplement: Supplementary file 1 — Supplementary Material 1 [file 12985_2025_2692_MOESM1_ESM.docx]

**Supplementary appendix**

***Table 1***. Diagnosis of acute cardiac events modified according to “German ICD-10”

| **ICD-10** | **Acute cardiac event** |  |  |
| --- | --- | --- | --- |
| I10.01 | Benigne essentielle Hypertonie: Mit Angabe einer hypertensiven Krise | I21.2 | Akuter transmuraler Myokardinfarkt an sonstigen Lokalisationen |
| I10.11 | Maligne essentielle Hypertonie: Mit Angabe einer hypertensiven Krise | I21.3 | Akuter transmuraler Myokardinfarkt an nicht näher bezeichneter Lokalisation |
| I10.91 | Essentielle Hypertonie, nicht näher bezeichnet: Mit Angabe einer hypertensiven Krise | I21.4- | Akuter subendokardialer Myokardinfarkt |
| I11.01 | Hypertensive Herzkrankheit mit (kongestiver) Herzinsuffizienz: Mit Angabe einer hypertensiven Krise | I21.40 | Akuter subendokardialer Myokardinfarkt, Typ-1-Infarkt |
| I11.91 | Hypertensive Herzkrankheit ohne (kongestive) Herzinsuffizienz: Mit Angabe einer hypertensiven Krise | I21.41 | Akuter subendokardialer Myokardinfarkt, Typ-2-Infarkt |
| I12.01 | Hypertensive Nierenkrankheit mit Niereninsuffizienz: Mit Angabe einer hypertensiven Krise | I21.48 | Sonstiger und nicht näher bezeichneter akuter subendokardialer Myokardinfarkt |
| I12.91 | Hypertensive Nierenkrankheit ohne Niereninsuffizienz: Mit Angabe einer hypertensiven Krise | I21.9 | Akuter Myokardinfarkt, nicht näher bezeichnet |
| I13.01 | Hypertensive Herz- und Nierenkrankheit mit (kongestiver) Herzinsuffizienz: Mit Angabe einer  hypertensiven Krise | I22.0 | Rezidivierender Myokardinfarkt der Vorderwand |
| I13.21 | Hypertensive Herz- und Nierenkrankheit mit (kongestiver) Herzinsuffizienz und Niereninsuffizienz:  Mit Angabe einer hypertensiven Krise | I22.1 | Rezidivierender Myokardinfarkt der Hinterwand |
| I15.01 | Renovaskuläre Hypertonie: Mit Angabe einer hypertensiven Krise | I22.8 | Rezidivierender Myokardinfarkt an sonstigen Lokalisationen |
| I15.11 | Hypertonie als Folge von sonstigen Nierenkrankheiten: Mit Angabe einer hypertensiven Krise | I22.9 | Rezidivierender Myokardinfarkt an nicht näher bezeichneter Lokalisation |
| I15.21 | Hypertonie als Folge von endokrinen Krankheiten: Mit Angabe einer hypertensiven Krise | I23.0 | Hämoperikard als akute Komplikation nach akutem Myokardinfarkt |
| I15.81 | Sonstige sekundäre Hypertonie: Mit Angabe einer hypertensiven Krise | I23.1 | Hämoperikard als akute Komplikation nach akutem Myokardinfarkt |
| I15.91 | Sekundäre Hypertonie, nicht näher bezeichnet: Mit Angabe einer hypertensiven Krise | I23.2 | Ventrikelseptumdefekt als akute Komplikation nach akutem Myokardinfarkt |
| I20.0 | Instabile Angina pectoris | I23.3 | Ruptur der Herzwand ohne Hämoperikard als akute Komplikation nach akutem Myokardinfarkt |
| I21.0 | Akuter transmuraler Myokardinfarkt der Vorderwand | I23.4 | Ruptur der Chordae tendineae als akute Komplikation nach akutem Myokardinfarkt |
| I23.5 | Papillarmuskelruptur als akute Komplikation nach akutem Myokardinfarkt | I40.8 | Sonstige akute Myokarditis |
| I23.8 | Sonstige akute Komplikationen nach akutem Myokardinfarkt | I40.9 | Akute Myokarditis, nicht näher bezeichnet |
| I24.8 | Sonstige Formen der akuten ischämischen Herzkrankheit | I41 | Myokarditis bei anderenorts klassifizierten Krankheiten |
| I24.9 | Akute ischämische Herzkrankheit, nicht näher bezeichnet | I47.0 | Ventrikuläre Arrhythmie durch Re-entry |
| I26.0 | Lungenembolie mit Angaben eines akuten Cor Pulmonale | I47.2 | Ventrikuläre Tachykardie |
| I30.0 | Akute unspezifische idiopathische Perikarditis | I49.0 | Kammerflattern und Kammerflimmern |
| I30.1 | Infektiöse Perikarditis | I50.00 | Primäre Rechtsherzinsuffizienz |
| I30.8 | Sonstige Formen der akuten Perikarditis | I50.01 | Sekundäre Rechtsherzinsuffizienz |
| I30.9 | akute Perikarditis, nicht näher bezeichnet | I50.04! | Rechtsherzinsuffizienz mit Beschwerden bei leichterer Belastung |
| I33.0 | Akute und subakute infektiöse Endokarditis | I50.05! | Rechtsherzinsuffizienz mit Beschwerden in Ruhe |
| I33.9 | Akute Endokarditis, nicht näher bezeichnet | I50.13 | Mit Beschwerden bei leichterer Belastung |
| I40.0 | Infektiöse Myokarditis | I50.14 | Mit Beschwerden in Ruhe |
| I40.1 | Isolierte Myokarditis | R57.0 | Kardiogener Schock |

***Table 2a*.** Patients characteristics by age groups.

| **Characteristics** | All swabs, n = 1756, excluding n = 8 viral co-infections, **p<0.05 = bold,** ** Mann-Withney U-test* | | | | | | | | | | | |
| --- | --- | --- | --- | --- | --- | --- | --- | --- | --- | --- | --- | --- |
|  | PCR-negative swabs | | RSV A and B | | Influenza A and B | | *p-value*  *(RSV vs. Influenza)* | | SARS-CoV-2 | | *p-value*  *(RSV vs. SARS-CoV-2)* | |
|  | n = 1287 | | n = 38 | | n = 147 | |  | | n = 284 | |  | |
|  | Age 18-59 y | Age ≥ 60y | Age 18-59 y | Age ≥ 60y | Age 18-59 y | Age ≥ 60y | Age 18-59 y | Age ≥ 60y | Age 18-59 y | Age ≥ 60y | Age 18-59 y | Age ≥ 60y |
| n | 451 (35.0) | 836 (65.0) | 6 (15.8) | 32 (84.2) | 83 (56.5) | 64 (43.5) |  |  | 59 (20.8) | 225 (79.2) |  |  |
| Males, n (%) | 232 (51.4) | 484 (57.9) | 4 (66.7) | 18 (56.3) | 43 (51.8) | 35 (54.7) | *0.484** | *0.885** | 40 (67.8) | 131 (58.2) | *0.974 ** | *0.833** |
| CCI, *score*  *(median; quartils)* | 0 (0;2) | 2 (1;4) | 0 (0;3) | 2.5 (1;3) | 0 (0;1) | 1 (0;3) | *0.903** | *0.055** | 0 (0;1) | 2 (0;3) | *0.938** | *0.061* |
| **Respiratory Support** |  |  |  |  |  |  |  |  |  |  |  |  |
| Oxygen only, n (%) | 81 (18.0) | 395 (47.2) | 0 | 24 (75.0) | 16 (19,3) | 34 (53.1) | *0.238** | ***0.040**** | 10 (16.9) | 108 (48.0) | *0.513** | ***0.004**** |
| nHFOT, n (%) | 6 (1.3) | 26 (3.1) | 0 | 2 (6.3) | 0 | 2 (3.1) | *1.000** | *0.472** | 1 (1.7) | 10 (4.4) | *0.956** | *0.651** |
| NIV, n (%) | 3 (0.7) | 60 (7.2) | 0 | 1 (3.1) | 2 (2.4) | 2 (3.1) | *0.702** | *1.000** | 0 | 12 (5.3) | *1.000** | *0.594** |
| Invasive  ventilation, n (%) | 12 (2.7) | 27 (3.2) | 0 | 0 | 3 (3.6) | 1 (1.6) | *0.638** | *0.480** | 1 (1.7) | 2 (0.9) | *0.956** | *0.593** |
| **Admission** |  |  |  |  |  |  |  |  |  |  |  |  |
| Inpatient, n (%) | 233 (51.7) | 709 (84.8) | 3 (50.0) | 28 (87.5) | 40 (48.2) | 54 (84.4) | *0.932 ** | *0.684** | 29 (49.2) | 210 (93.3) | *0.974** | *0.239** |
| ICU-admission, n (%) | 20 (4.4) | 86 (10.3) | 0 | 0 | 7 (8.4) | 6 (9.4) | *0.461** | *0.075** | 1 (1.7) | 11 (4.9) | *0.956** | *0.202** |
| Length of stay, only inpatients, *days (median; quartils)* | 7 (4;13) | 9 (5;17) | 6 (2;6) | 9 (6;15) | 7 (2;11) | 8 (5;12) | *0.423** | *0.184** | 7 (3;9) | 8 (4;13) | *0.286** | *0.074** |
| **Outcome** |  |  |  |  |  |  |  |  |  |  |  |  |
| Death, n (%) | 9 (2.0) | 79 (9.4) | 0 | 3 (9.4) | 2 (2.4) | 2 (3.1) | *0.702** | *0.196** | 1 (1.7) | 15 (6.7) | *0.956** | *0.575** |
| Acute cardiac  event, n (%) | 26 (5.8) | 227 (27.2) | 1 (16.7) | 11 (34.4) | 3 (3.6) | 10 (15.6) | *0.138** | ***0.037**** | 3 (5.1) | 44 (19.6) | *0.650** | *0.056** |

***Table 2b.*** Patients characteristics by comorbidities.

| Characteristics | All swabs, n = 1756, excluding n = 8 viral co-infections, **p<0.05 = bold,** ** Mann-Whitney U-test* | | | | | | | | | | | |
| --- | --- | --- | --- | --- | --- | --- | --- | --- | --- | --- | --- | --- |
|  | PCR-negative swabs | | RSV A and B | | Influenza A and B | | *p-value*  *(RSV vs. Influenza)* | | SARS-CoV-2 | | *p-value*  *(RSV vs. SARS-CoV-2)* | |
|  | n = 1287 | | n = 38 | | n = 147 | |  | | n = 284 | |  | |
|  | CCI = 0 | CCI ≥ 1 | CCI = 0 | CCI ≥ 1 | CCI = 0 | CCI ≥ 1 | CCI = 0 | CCI ≥ 1 | CCI = 0 | CCI ≥ 1 | CCI = 0 | CCI ≥ 1 |
| n (%) | 457 (35.5) | 830 (64.5) | 8 (21.1) | 30 (78.9) | 70 (47.6) | 77 (52.3) |  |  | 102 (35.9) | 182 (64.1) |  |  |
| Age, y *(median; quartils)* | 53 (34;72) | 72 (61;82) | 59.5 (37;77) | 81 (71;86) | 45.5 (34;68) | 63 (43;82) | *0.429** | ***<0.001*** | 68.5 (41;83) | 81 (70;85) | *0.277** | *0.359* |
| Males, n (%) | 220 (48.1) | 496 (59.8) | 5 (62.5) | 17 (56.7) | 36 (51.4) | 42 (54.5) | *0.555** | *0.844** | 58 (56.9) | 113 (62.1) | *0.757** | *0.573** |
| **Respiratory Support** |  |  |  |  |  |  |  |  |  |  |  |  |
| Oxygen only, n (%) | 66 (14.4) | 410 (49.4) | 1 (12.5) | 23 (76.7) | 8 (11.4) | 42 (54.5) | *0.929** | ***0.036**** | 22 (21.6) | 96 (52.7) | *0.545** | ***0.015**** |
| nHFOT, n (%) | 5 (1.1) | 27 (3.3) | 0 | 2 (6.7) | 0 | 2 (2.6) | *1.000** | *0.321** | 1 (1.0) | 10 (5.5) | *0.779** | *0.797** |
| NIV, n (%) | 4 (0.9) | 59 (7.1) | 0 | 1 (3.3) | 0 | 4 (5.2) | *1.000** | *0.683** | 1 (1.0) | 11 (6.0) | *0.779** | *0.553** |
| Invasive  ventilation, n (%) | 8 (1.8) | 31 (3.7) | 0 | 0 | 0 | 4 (5.2) | *1.000** | *0.205** | 0 | 3 (1.6) | *1.000** | *0.480** |
| **Admission** |  |  |  |  |  |  |  |  |  |  |  |  |
| Inpatient, n (%) | 180 (39.4) | 762 (91.8) | 3 (37.5) | 28 (93.3) | 21 (30.0) | 73 (94.8) | *0.665** | *0.767** | 62 (60.8) | 177 (97.3) | *0.199** | *0.267** |
| ICU-admission, n (%) | 4 (0.9) | 112 (13.5) | 0 | 0 | 2 (2.9) | 11 (14.3) | *0.630** | ***0.030**** | 0 | 12 (6.6) | *1.000** | *0.149** |
| Length of stay, only inpatients, *days (median; quartils)* | 6 (3;10) | 9.5 (5;17) | 8 (2;8) | 8.5 (6;15) | 5 (3;8) | 8 (4;12) | *0.742** | *0.237** | 6.5 (3;10) | 8 (4;13) | *0.918** | *0.197** |
| **Outcome** |  |  |  |  |  |  |  |  |  |  |  |  |
| Death, n (%) | 8 (1.8) | 80 (9.6) | 0 | 3 (10.0) | 0 | 4 (5.2) | *1.000** | *0.396** | 3 (2.9) | 13 (7.1) | *0.624** | *0.584** |
| Acute cardiac  event, n (%) | 21 (4.6) | 232 (28.0) | 0 | 12 (40.0) | 0 | 13 (16.9) | *1.000** | ***0.012**** | 5 (4.9) | 42 (23.1) | *0.523** | ***0.049**** |
